# Lateral inhibition by Martinotti interneurons is facilitated by cholinergic inputs in human and mouse neocortex

**DOI:** 10.1038/s41467-018-06628-w

**Published:** 2018-10-05

**Authors:** Joshua Obermayer, Tim S. Heistek, Amber Kerkhofs, Natalia A. Goriounova, Tim Kroon, Johannes C. Baayen, Sander Idema, Guilherme Testa-Silva, Jonathan J. Couey, Huibert D. Mansvelder

**Affiliations:** 1 Department of Integrative Neurophysiology, Center for Neurogenomics and Cognitive Research, Neuroscience Campus, VU University Amsterdam, De Boelelaan 1085, Amsterdam, 1081 HV The Netherlands; 2 Department of Neurosurgery, Neuroscience Campus Amsterdam, VU University Medical Center Amsterdam, De Boelelaan 1117, Amsterdam, 1081 HV The Netherlands; 3 Present Address: MRC Centre-Developmental Neurobiology, King’s college London, London, WC2R 2LS UK; 4 Present Address: Department of Chemistry and Chemical Biology, Harvard University, Cambridge, MA 02138 USA; 5 Present Address: Department of Neurobiology, University of Pittsburgh, Pittsburgh, PA 15261 PA USA

## Abstract

A variety of inhibitory pathways encompassing different interneuron types shape activity of neocortical pyramidal neurons. While basket cells (BCs) mediate fast lateral inhibition between pyramidal neurons, Somatostatin-positive Martinotti cells (MCs) mediate a delayed form of lateral inhibition. Neocortical circuits are under control of acetylcholine, which is crucial for cortical function and cognition. Acetylcholine modulates MC firing, however, precisely how cholinergic inputs affect cortical lateral inhibition is not known. Here, we find that cholinergic inputs selectively augment and speed up lateral inhibition between pyramidal neurons mediated by MCs, but not by BCs. Optogenetically activated cholinergic inputs depolarize MCs through activation of ß2 subunit-containing nicotinic AChRs, not muscarinic AChRs, without affecting glutamatergic inputs to MCs. We find that these mechanisms are conserved in human neocortex. Cholinergic inputs thus enable cortical pyramidal neurons to recruit more MCs, and can thereby dynamically highlight specific circuit motifs, favoring MC-mediated pathways over BC-mediated pathways.

## Abstract (web summary)

Parvalbumin and somatostatin expressing interneurons mediate lateral inhibition between cortical neurons. Here the authors report the mechanisms by which acetylcholine from the basal forebrain selectively augments lateral inhibition via Martinotti cells and show that this is conserved in humans.

## Introduction

Inhibition of pyramidal neurons by GABAergic interneurons is essential for cortical computation. Several circuit motifs have been identified by which interneurons shape cortical signal propagation, among which are feedforward inhibition, feedback inhibition and disinhibition^1,2^. In each of these motifs, several distinct types of interneurons can be involved. For instance, lateral inhibition, a form of feedback inhibition generated by activity in local circuits of pyramidal neurons and interneurons, can be mediated by parvalbumin (PV)-positive fast-spiking basket cells as well as somatostatin (SOM)-positive interneurons^3–5^. Because of the profound difference in projection targets on pyramidal neuron dendrites between PV and SOM axons, whereby PV neurons innervate perisomatic regions and SOM neurons generally target distal dendrites, lateral inhibition by PV neurons may be more involved in rapidly silencing action potential firing in neighboring pyramidal neurons, while lateral inhibition through SOM neurons will control synaptic integration, burst firing and dendritic regenerative phenomena^1,4,6,7^. What the precise impact will be of lateral inhibition by a given interneuron type at any point in time will depend among other things on neuromodulatory conditions, but this is poorly understood. Both PV and SOM interneurons are modulated by various neurotransmitters^1^ and in particular SOM interneurons are strongly modulated by acetylcholine^8–13^. The cortex receives cholinergic inputs mainly from the basal forebrain^14,15^. How cholinergic inputs affect lateral inhibition is not known. It is also not known whether lateral inhibition between pyramidal neurons exists in human neocortical circuits. Here, we address these issues.

Both PV-positive basket cells (BCs) and SOM-positive Martinotti cells (MCs) form disynaptic inhibitory microcircuits with pyramidal neurons that enable them to alter activity of surrounding pyramidal cells^5,16^. A single pyramidal cell can activate BCs and MCs when spiking at high frequencies, which in turn leads to lateral inhibition of neighboring pyramidal cells^5^. Whereas only a subgroup of BCs show a response to acetylcholine, it induces strong action potential firing in MCs via both muscarinic and nicotinic acetylcholine receptors^8–12^, which has been implied to be involved in cholinergic modulation of cortical function^10,17–19^. Here, we investigate the mechanisms by which cholinergic inputs from the basal forebrain affect fast and delayed disynaptic inhibition between pyramidal cells (PCs). In simultaneous recordings from synaptically connected neocortical neurons we find that only delayed lateral inhibition via MCs is modulated by cholinergic inputs, while fast lateral inhibition via BCs is not. We demonstrate that somatic depolarization of MCs, rather than changes in synaptic strength, induced by endogenously released ACh from basal forebrain projections augments lateral inhibition in both supragranular and infragranular layers in both the medial prefrontal cortex (mPFC) and the somatosensory cortex. In addition, we show that lateral inhibition is evolutionary conserved in the human neocortex and is facilitated by ACh through similar mechanisms.

## Results

### Delayed lateral inhibition is selectively enhanced by basal forebrain cholinergic inputs

**Fig. 1:**
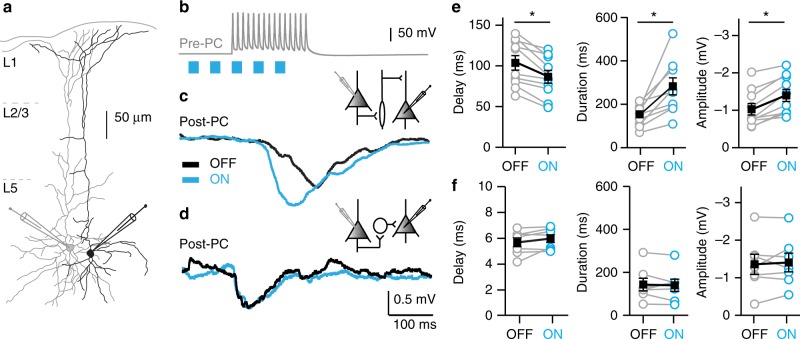
Cholinergic inputs selectively enhance delayed lateral inhibition. **a** Digital reconstruction of two Biocytin-filled layer 5 (L5) pyramidal neurons in coronal slices of the mouse medial prefrontal cortex (mPFC). **b** Example trace of APs induced in a presynaptic L5 pyramidal neuron (Pre-PC). Gray trace: the Pre-PC cell is stimulated to fire 15 APs at 100 Hz. The presynaptic AP train coincides with optogenetic activation of ChR2-expressing cholinergic fibers with five short blue light flashes at 25 Hz, 100 ms preceding the first AP (blue bars). **c** Example trace of a delayed disynaptic inhibitory response in the postsynaptic pyramidal cell (Post-PC) in absence (OFF, black trace) or presence (ON, blue trace) of endogenous ACh release. **d** As **c** but in contrast a typical fast disynaptic inhibitory response in the Post-PC. **e** Summary charts showing that activation of cholinergic projections shortens the onset delay, increases the time course and amplitude of delayed disynaptic inhibition in mPFC L5 (Delay: light OFF 104 ± 9 ms, light ON 87 ± 8 ms, paired *t*-test, two-tailed, *p* = 0.0009, *t* = 4.8, df = 9; Duration: light OFF 141 ± 16 ms, light ON 274 ± 41 ms, Wilcoxon signed-rank test, *p* = 0.002; Amplitude: light OFF 1.01 ± 0.16 mV, light ON 1.40 ± 0.19 mV; paired *t*-test, two-tailed, *p* = 0.0002, *t* = 5.9, df = 9; *n* = 10, mean ± s.e.m.). **f** As **e**, showing that in mPFC L5 fast disynaptic lateral inhibition is not affected by optogenetic activation of cholinergic projections (Delay: light OFF 5.58 ± 0.356 ms, light ON 5.88 ± 0.3 ms, *p* = 0.1249, paired *t*-test, two-tailed, *t* = 1.783, df = 6; Duration: light OFF 140 ± 29.12 ms, light ON 136.9 ± 28,3 ms, *p* = 0.7733, paired *t*-test, two-tailed, *t* = 0.3014, df = 6; Amplitude: light OFF 1.316 ± 0.26 mV, light ON 1.36 ± 0.24 mV, *p* = 0.6425, paired *t*-test, two-tailed, *t* = 0.4885, df = 6; *n* = 7; mean ± s.e.m.)

Pyramidal neurons in the neocortex can inhibit neighboring pyramidal cells (PCs) by feedforward activation of inhibitory interneurons^5,16^. This process of disynaptic inhibition has been observed in several cortical areas and in different cortical layers^1,3^. As the majority of interneurons in the cortex express acetylcholine receptors (AChRs)^9,11,20–23^, we tested whether acetylcholinergic (ACh) inputs that come mainly from the basal forebrain (BF) modulate disynaptic lateral inhibition between pyramidal neurons. We recorded from up to four pyramidal cells simultaneously in layer 2/3 (L2/3) or L5 in acute brain slices of the mPFC or the somatosensory cortex (Fig. 1a). To recruit disynaptic inhibitory loops, presynaptic pyramidal cells (Pre-PC) were triggered to fire 15 action potentials (APs) at 100 Hz, which induced stereotypic fast or delayed inhibitory postsynaptic responses (IPSPs) in pyramidal cells (Post-PC), as reported by Silberberg et al.^5^ and Kapfer et al.^3^ (Fig. 1b–d, Pre-PC and Post-PC traces). Fast and delayed inhibitory responses were identified with K-means cluster analysis on onset latency and latency to peak (Supplementary Figure 1). Cholinergic projections were stimulated optogenetically by blue light activation of channelrhodopsin (ChR2) expressed by ChAT-positive neurons (see Methods). Since cholinergic neurons of the basal forebrain fire in bursts during wakefulness^24,25^, and to approximate physiologically relevant rates of ACh release, we used five blue light pulses with a frequency of 25 Hz to release endogenous ACh. Previous results showed that optogenetically released ACh can induce feedforward inhibitory responses in pyramidal neurons^26^. In a few cases, we also observed feedforward inhibitory events following optogenetically triggered ACh release. These recordings were excluded from analysis. One hundred milliseconds after the first blue light stimulus, the Pre-PC cells were triggered to fire 15 APs with a frequency of 100 Hz (Fig. 1b). Optogenetic activation of cholinergic inputs resulted in a shorter onset latency of delayed, but not fast disynaptic IPSPs in postsynaptic L5 pyramidal cells of the mPFC (Fig. 1c, d). Furthermore, optogenetic stimulation of cholinergic inputs altered the kinetics of delayed disynaptic inhibition, increasing both the time course (Fig. 1c, e) and the amplitude (Fig. 1c, e) of delayed disynaptic IPSPs. In contrast, cholinergic inputs did not affect the duration (Fig. 1d, f) or amplitude (Fig. 1d, f) of fast disynaptic inhibition. In L2/3 of the primary sensory cortex (S1) we observed qualitatively and quantitatively similar modulation of delayed disynaptic IPSPs following optogenetic activation of cholinergic projections (see Supplementary Figure 2 below). This shows that cholinergic inputs to the neocortex modulate delayed but not fast lateral inhibition between pyramidal neurons.

**Fig. 2:**
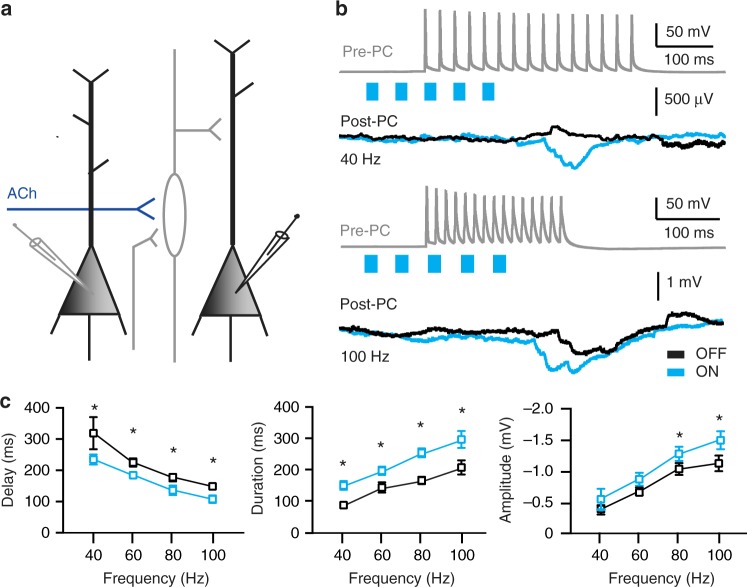
ACh enhances lateral inhibition independent of presynaptic firing rate. **a** Schematic representation of the experiment with the presynaptic (Pre-PC) and postsynaptic (Post-PC) pyramidal neurons, as well as cholinergic projections to an interneuron. **b** Example traces of an AP train fired by a presynaptic L5 mPFC pyramidal neuron and the resulting lateral inhibition received by the postsynaptic pyramidal neurons. Top trace: Current is injected in the Pre-PC neuron to induce trains of 15 AP with different frequencies (40, 60, 80, 100 Hz). The presynaptic stimulation is combined with or without five short blue light flashes at 25 Hz starting 100 ms before the first AP (blue bars) for optogenetic activation of cholinergic projections. Bottom trace: Example traces of delayed disynaptic inhibitory response in Post-PC either in absence (OFF, black trace) or presence (ON, blue trace) of cholinergic projection activation. **c** Summary charts showing that activation of cholinergic projections shortens the onset delay (F_(3, 22)_ = 22.80, One-way ANOVA, *p* < 0.05), increase the time duration (F_(3, 22)_, = 11,60, One-way ANOVA, *p* < 0.01) and amplitude (F_(3, 22)_ = 11.53, One-way ANOVA, *p* < 0.05)of the delayed disynaptic IPSP independent from the firing frequency of the Pre-PC (*n* = 7). The ratio of the cholinergic facilitation is not dependent from different frequencies (Delay: F_(3, 22)_ = 1.622, One-way ANOVA, *p* = 0.2105; Duration: F_(3, 22)_ = 1.466, One-way ANOVA, *p* = 0.2510; Amplitude: F_(3, 22)_ = 1260, One-way ANOVA, *p* = 0. 3125; *n* = 7, mean ± s.e.m.)

Previous work has shown that induction and kinetics of lateral inhibitory postsynaptic potentials correlate with firing frequencies of presynaptic pyramidal neurons^5^. We asked whether the modulation of lateral inhibition by cholinergic projections is less pronounced when presynaptic pyramidal cells (Pre-PC) are firing trains of APs at lower frequencies. To address this question, we stimulated presynaptic pyramidal neurons to fire trains of 15 APs at different frequencies (40, 60, 80, 100 Hz) and simultaneously recorded inhibitory responses in postsynaptic pyramidal cells (Fig. 2a, b). Independent from the firing frequency of presynaptic pyramidal neurons, optogenetic activation of cholinergic projections led to a shorter onset delay (Fig. 2c), an increased duration (Fig. 2c) and a larger amplitude (Fig. 2c) of disynaptic IPSPs. Facilitation by cholinergic projections of onset (Fig. 2c), duration (Fig. 2c) and amplitude (Fig. 2c) of disynaptic inhibition was not different between applied frequencies. These results indicate that cholinergic projections facilitate lateral inhibition independent of pyramidal neuron firing frequencies.

**Fig. 3:**
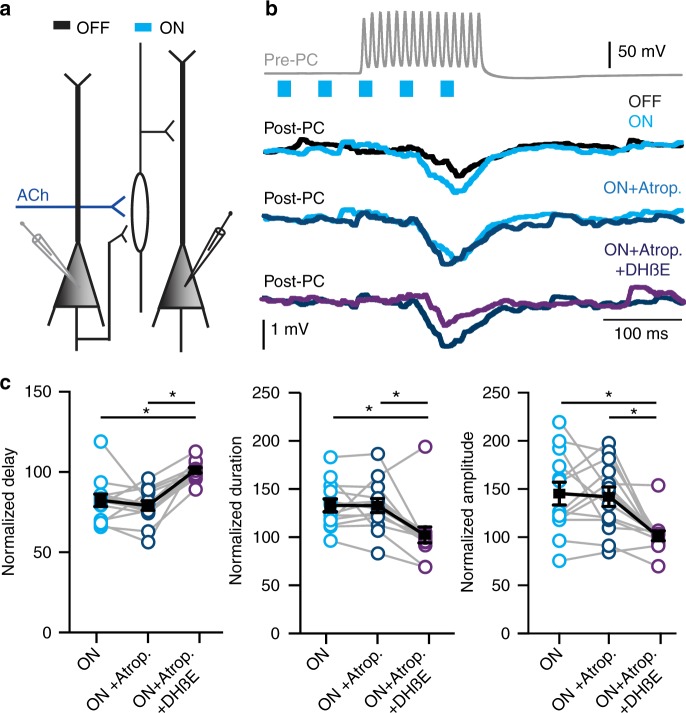
Cholinergic inputs facilitate lateral inhibition via heteromeric nAChRs. **a** Recording from two layer 5 pyramidal neurons in the mPFC showing disynaptic inhibition. Top trace: Example trace of AP firing by the Pre-PC (15 AP at 100 Hz) combined with or without blue light for optogenetic activation of cholinergic projections (blue bars), starting 100 ms before the electrical stimulation. Second trace: Example trace of an inhibitory response in the Post-PC neuron in absence (OFF, black trace) or presence (ON, blue trace) of cholinergic projection activation. Third trace: As second trace, in presence of atropine (400 mM). Fourth trace: As second trace, in presence of atropine (400 mM) and DHßE (10 µM). **b** Summary chart showing that ACh shortens the onset delay of lateral inhibition. Modulation of lateral inhibition by cholinergic projection activation is unaffected by mAChR antagonist atropine. Modulation of lateral inhibition by cholinergic projection activation is blocked by DHßE, an antagonist of heteromeric nAChRs (F_(12,24)_ = 2.068, *p* < 0.01; One-way ANOVA, *n* = 13, mean ± s.e.m.). **c** As in **b** showing that the increase in duration of lateral inhibition does depend on activation of heteromeric nAChRs but not mAChRs ((F_(12,24)_ = 2.355, *p* = 0.0082; One-way ANOVA, *n* = 13, mean ± s.e.m.). **d** As in **b** and **c** showing that the cholinergic modulation of the amplitude of lateral inhibition depends on activation of heteromeric nAChRs (F_(12,24)_ = 2.288, *p* = 0.0012; One-way ANOVA, *n* = 13, mean ± s.e.m.)

The neuromodulator ACh can shape neuronal circuits by activation of muscarinic and nicotinic acetylcholine receptors (mAChRs, nAChRs, respectively), and cortical interneurons have been shown to express both types of receptors^8–12^. To determine whether the modulation of delayed disynaptic inhibitory loops by cholinergic projections is mediated by mAChRs, nAChRs, or both, we applied the mAChR antagonist atropine followed by the application of both the heteromeric nAChR antagonist DHßE and atropine (Fig. 3a, b). Atropine (400 nM) did not affect the modulation of disynaptic inhibition by optogenetic activation of cholinergic projections (Fig. 3b, c). In the presence of atropine, optogenetic activation of cholinergic projections sped up the onset latency and the amplitude of the IPSPs in the postsynaptic pyramidal cells similar to control conditions (Fig. 3b, c). In contrast to atropine, the nAChR antagonist DHßE (10 µM) abolished the modulatory effects of endogenous ACh on onset delay, duration, and amplitude (Fig. 3b, c). These pharmacological manipulations show that modulation of disynaptic inhibitory loops by cholinergic inputs is mediated almost exclusively by heteromeric nAChRs and not by mAChRs.

Cholinergic projections from the basal forebrain have been shown to co-release other neurotransmitters, such as GABA^27^. To exclude potential involvement of other neurotransmitters, we tested whether bath application of ACh in the presence of atropine would modulate disynaptic inhibition in a similar fashion as optogenetic activation of cholinergic projections. Upon application of ACh (1 mM, atropine 400 nM) we observed a decrease in the onset delay of the disynaptic IPSPs, similar to optogenetic activation of cholinergic projections (Supplementary Figure 2B) as well as an increase in the time course of disynaptic IPSPs (Supplementary Figure 2C). After 15 min washout of ACh, the onset delay and duration of the IPSPs returned to baseline before ACh application (Supplementary Figure 2B, C). IPSP amplitude was not modulated by bath application of ACh (Supplementary Figure 2D). Taken together, these results show that cholinergic inputs from the basal forebrain speed up the action and increase the amplitude of lateral inhibition between neocortical pyramidal neurons via activation of heteromeric nAChRs.

### Cholinergic inputs directly depolarize Martinotti Cells

**Fig. 4:**
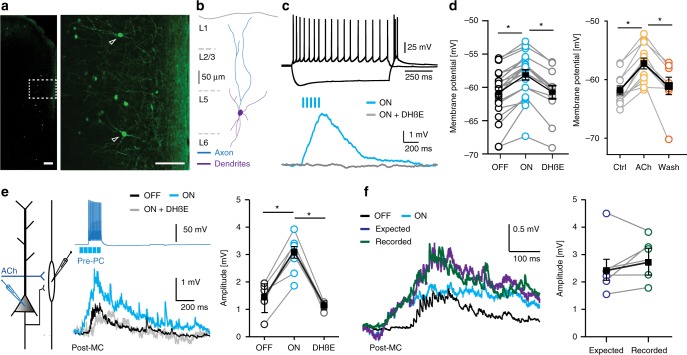
Cholinergic inputs depolarize Martinotti cells. **a** GFP-labeled SOM + interneurons in a GIN mouse (scale bar 200 µm). **b** Digital reconstruction of a GFP-expressing MC. **c** Top trace: Action potential profile of a L5 MC in a GIN/Chat-ChR2-EYFP mouse in response to somatic current injection (+100 pA and −150 pA). Bottom trace: Example trace (Blue trace) of a nAChR-mediated response in a L5 MC in the mPFC. The postsynaptic response was blocked by DHßE (10 µM, gray trace). **d** Left: Summary chart showing the maximum amplitude of nAChR-EPSPs in L5 MCs in the mPFC evoked by optogenetic triggered ACb release (light OFF −60.68 ± 0.82 mV, light ON −58.00 ± 0.76 mV, DHßE −60.60 ± 1.00 mV, F_(2, 52)_ = 3.488, one-way ANOVA, *p* = 0.038, *n* = 23; mean ± s.e.m.). Right: Summary chart indicating the depolarization of L2/3 MCs of S1 by application of ACh (1 mM) (Ctrl. −61.8 ± 0.75 mV, ACh −57.2 ± 0.94 mV, wash −61.0 ± 1.48 mV, paired *t*-test, two-tailed, *p* = 0.0005, *t* = 8.288, df = 11, *n* = 12). **e** Right: Setup of the experiment. Middle: Example traces of synaptically connected pre-PC and postsynaptic MC (Post-MC). Middle top: Pre-PC fired a train of 15 APs at 100 Hz. Optogenetic ACh release was induced by five light pulses at 25 Hz starting 100 ms preceding the first AP. Middle bottom: Postsynaptic responses recorded in a mPFC L5 Post-MC in absence (Black trace) or presence of optogenetic triggered ACh release (Blue trace). The potentiation that is induced by ACh is blocked by DHßE (Gray trace). Right: Summary plot. The combination of glutamatergic EPSPs from the PC and cholinergic excitatory input leads depolarizes the membrane potential (light OFF 1.54 ± s.e.m. mV, light ON 3.30 ± s.e.m. mV). This is blocked by application of DHßE (1.07 mV, One-way ANOVA F_(2,13)_ = 16.81, *p* = 0.0002, *n* = 6). **f** Left: Example trace of a glutamatergic EPSP (Black trace) and nAChR-mediated (Blue trace) EPSPs. The co-occurring of glutamatergic and cholinergic EPSPs (Green trace) leads to larger depolarization. Right: summation of single glutamatergic and nAChR-mediated EPSPs (Expected value, Purple trace, 2.54 mV ± s.e.m.) did not differ from the recorded combined EPSP (2.88 mV ± s.e.m., *p* = 0.2766, paired *t*-test, two-tailed, *t* = 1.221, df = 5, *n* = 6; mean ± s.e.m.)

Previous studies have shown that Martinotti Cells (MCs) in the neocortex mediate lateral inhibition through disynaptic loops between pyramidal neurons^3–5,28^. In addition, MCs express a mixed population of somatic α7 and non-α7 nAChRs, but nicotinic receptor currents are dominated by heteromeric nAChRs containing ß2 subunits^9,11^. To address the question whether a change in MC activity caused by ACh release is responsible for the modulation of disynaptic inhibition between pyramidal neurons, we recorded from GFP-expressing MCs in the “GIN” mouse line^29^ and either optogenetically activated cholinergic projections or bath applied ACh (1 mM) (Methods; Fig. 4a). For optogenetic activation of cholinergic projections, acute mPFC slices of CHAT-ChR2 mice crossed with “GIN” mice were used and five short (10 ms duration) blue light pulses (25 Hz) were applied. In the experiments using ACh bath application, ACh was washed in for 15 min in the presence of atropine (400 nM). Optogenetic activation of cholinergic projections resulted in a depolarization of the membrane potential in the MCs (Fig. 4c, d). During the five light pulses at 25 Hz, the cholinergic postsynaptic depolarization in the Martinotti cell continued to increase (Fig. 4c). The depolarization ended only after the final light pulse. This suggests that with each light pulse additional ACh was released, and may suggest that cholinergic fibers were activated by each light pulse in the five pulse train. The depolarization was completely abolished by bath application of DHßE (10 µM) (Fig. 4c, d). Bath application of ACh (1 mM) depolarized MCs to a similar degree (Fig. 4d), which was reversed following washout (Fig. 4d, *p* < 0.001). These results show that both in S1 L2/3 and mPFC L5, ACh from projections depolarizes MCs by activation of postsynaptic heteromeric nAChRs.

Next, we simultaneously recorded from synaptically connected pyramidal-Martinotti cell pairs, and tested whether MC depolarization by cholinergic projections linearly summates with the depolarizations induced by synaptic inputs received from presynaptic pyramidal cells (Pre-PC, Fig. 4e). Excitatory postsynaptic potentials (EPSPs) received by MCs in response to AP firing of presynaptic pyramidal cells were compared with EPSPs that co-occurred with optogenetic activation of cholinergic projections. Optogenetic activation of cholinergic projections occurred 100 ms before the onset of AP firing of presynaptic pyramidal cells. MC depolarizations were strongly increased by combined optogenetic activation of cholinergic projections and EPSPs received from Pre-PCs (Fig. 4e). The nAChR antagonist DHßE blocked the ACh induced increase of the depolarization of the MC (Fig. 4e). To determine whether these depolarizations summate linearly, we calculated the linear sum of the depolarization induced by the EPSPs and cholinergic inputs separately (Fig. 4f), and compared this with the recorded depolarization when these events occurred simultaneously. We observed no significant difference between the recorded (green trace) and expected amplitude (purple trace) of depolarizations (Fig. 4f). These findings indicate that depolarizations by cholinergic inputs and synaptic EPSPs summate linearly to depolarize MCs.

### ACh does not affect synaptic strength between pyramidal and Martinotti cells

**Fig. 5:**
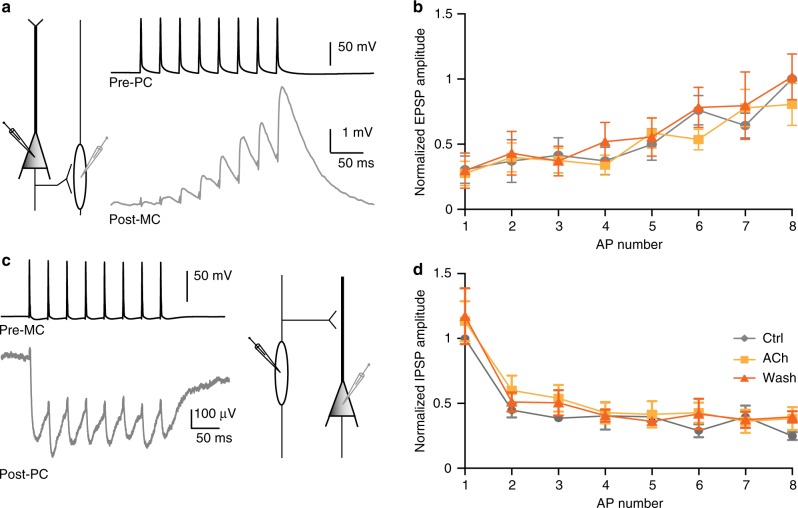
Cholinergic inputs do not affect synaptic strength between PCs and MCs. **a** Left: schematic representation of the simultaneous recording of a presynaptic pyramidal cell (Pre-PC) and a postsynaptic Martinotti cell (Post-MC). Example trace recorded from a PC-Pre cell injected with current to evoke 8 APs at a frequency of 30 Hz (Black trace) and the EPSPs in the Post-MC cell (Gray trace). **b** Summary plot of the normalized amplitude of EPSPs recorded in post-MCs. The amplitude was normalized to the last EPSP. Bath application and washout of ACh (1 mM) did not alter synaptic strength between the Pre-PC and Post-MC (F_(2, 21)_ = 0.2511, *p* = 0.7802, One-Way ANOVA; *n* = 6, mean ± s.e.m.). **c** Simultaneous recording of a presynaptic Martinotti cell (Pre-MC) and a postsynaptic pyramidal cell (Post-PC). A 30 Hz AP train was induced in the Pre-MC cell (Black trace) that induced a series of IPSPs in the Post-PC cell (Gray trace). **d** Summary plot showing the normalized amplitude of the IPSP in the Post-PC cell. The amplitude is normalized to the first IPSP. The amplitude of the IPSP is not changed in the presence of ACh (1 mM) or during ACh washout (F_(2,21)_ = 0.2705, *p* = 0.7656; One-way ANOVA; *n* = 7, mean ± s.e.m.)

Somatic depolarization of MCs may be sufficient to explain cholinergic modulation of lateral inhibition between pyramidal neurons. However, nAChRs can also be expressed on presynaptic terminals directly affecting neurotransmitter release and synaptic strength^30–32^. In recordings from synaptically connected pyramidal neurons and MCs in L2/3 of the somatosensory cortex, we tested whether ACh affected synaptic strength between pyramidal neurons and MCs (Fig. 5a, c). Presynaptic pyramidal neurons were driven with current pulses to trigger 8 APs at a frequency of 30 Hz, a frequency at which postsynaptic MCs are unlikely to fire APs, and recorded EPSPs in post-MCs in the presence or absence of ACh. We observed no difference in EPSP amplitudes, kinetics or facilitation of EPSPs in postsynaptic MCs between control, ACh wash-in and washout conditions (Fig. 5b,). Similarly, the IPSP amplitudes recorded in postsynaptic pyramidal cells that were induced by presynaptic MC stimulation were not significantly affected by ACh application (Fig. 5d). Next, we investigated whether endogenous released ACh has an effect on the strength of the synaptic connection between pyramidal neurons and MCs in L5 of the mPFC. For this, we electrically excited the presynaptic pyramidal neuron to fire 15 APs with a frequency of 100 Hz and triggered simultaneously ACh release from cholinergic fibers by applying five blue light pulses at 25 Hz starting 100 ms before the electrical stimulation (Supplementary Figure 3A). Combining presynaptic electrical stimulation with optogenetic induced ACh release did not affect the synaptic strength between the pyramidal neurons and MCs (Supplementary Figure 3B). These data indicate that augmentation of disynaptic inhibitory loops by ACh is not due to altered synaptic strength (or efficacy) between pyramidal and MCs, nor due to changes in the release machinery that would affect the time course of IPSP depression and EPSP facilitation.

### Cholinergic inputs advance and prolong Martinotti cell AP firing

**Fig. 6:**
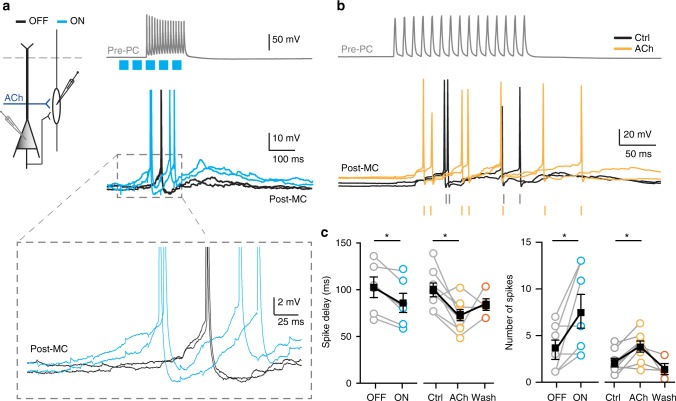
Cholinergic inputs facilitate AP firing by Martinotti cells. **a** Simultaneous recording from a presynaptic pyramidal (Pre-PC) and a postsynaptic Martinotti cell (Post-MC) in L5 of the mPFC. A 100 Hz AP train by the Pre-PC cell induces AP firing in the Post-MC cell (Black trace, OFF condition). Cholinergic inputs are optogenetically activated by five short blue light pulses at 25 Hz preceding the first induced AP by 100 ms (Blue trace). Simultaneous stimulation of the pre-PC and cholinergic inputs leads to a shorter onset delay and increased number of spikes in the MC-Post cell (Blue trace). However, we observed no change of the presynaptic membrane potential following activation of cholinergic fibers (light OFF −69.5 ± 4.47 mV, light ON 68.89 ± 3.60 mV, paired *t*-test, two-tailed, *p* = 0.536; *t* = 0.6406, df = 10, *n* = 11, mean ± s.e.m.). **b** Simultaneous recordings of a PC and MC recorded in L2/3 of the somatosensory cortex (S1). Representative traces of Post-MC APs in presence or absence of ACh (1 mM) (orange and black traces). Bath application of ACh did not lead to a depolarization of the presynaptic pyramidal neuron (Ctrl. −59.85 ± 5.422 mV, ACh −59.56 ± 5.067 mV, paired *t*-test, two-tailed, *p* = 0.3904; *t* = 0.8729, df = 27, *n* = 28). **c** Left: Summary plot of onset delay of the first AP. Right: the number of APs in the Post-MC cell. Optogenetic activation as well as bath application of ACh leads to a significant shortening of the onset delay time of Post-MC APs (light OFF 102.1 ± 10.79 ms, light ON 85.73 ± 10.18 ms, paired *t*-test, two-tailed, *p* = 0.026, *t* = 3.131, df = 5, *n* = 6; Ctrl. 99 ± 1 ms, ACh 69 ± 1 ms, paired *t*-test, two-tailed, *p* = 0.01, *t* = 3.4, df = 7, *n* = 8) and increase in number of APs during lateral inhibition (light OFF 3.429 ± 0.9724 APs, light ON 7.571 ± 1.744 APs, paired *t*-test, *p* = 0.042, *t* = 2.573, df = 6, *n* = 7; Ctrl. 2.1 ± 0.4 APs, ACh 3.8 ± 0.6 APs, paired *t*-test, two-tailed, *p* = 0.0045, *t* = 4.1, df = 7, *n* = 8). However, we observed no change of the AP threshold potential (light OFF −50.08 ± 4.127 mV, light ON 50.23 ± 4.562 mV, paired *t*-test, two-tailed, *p* = 0.82; *t* = 0.2348, df = 7, *n* = 8), or of the frequency of AP firing (light OFF 20.76 ± 10.28 Hz, light ON 20.97 ± 8.938 Hz, paired *t*-test, two-tailed, *p* = 0.8656; *t* = 0.1842, df = 3, *n* = 4)

Since depolarization of MCs is the most likely mechanism by which cholinergic projections modulate lateral inhibition between pyramidal cells, we tested whether cholinergic projections alter action potential firing of MCs in response to activation of presynaptic pyramidal cells. In simultaneous recordings from synaptically connected presynaptic pyramidal cells (Pre-PC) and MCs (Post-MC) in the mPFC, we tested whether ACh modulates the delay, number, and frequency of APs in MCs. Similar to previous experiments, presynaptic pyramidal cells were driven to fire 15 APs at 100 Hz and APs were recorded in the postsynaptic MCs (Fig. 6a, b example traces). Optogenetic activation of cholinergic projections was induced by five blue light pulses at 25 Hz starting 100 ms before the presynaptic pyramidal neuron stimulation. Only recordings were included for analysis in which the postsynaptic MC reliably fired APs in all experimental conditions (Fig. 6a, b). Combining presynaptic stimulation with light-evoked activation of cholinergic projections resulted in shortening of the latency to post-MC AP firing (Fig. 6a, c) and an increased number of APs in Post-MCs compared to Pre-PC stimulation alone without cholinergic projection activation (Fig. 6a, c). Similarly, bath application of ACh in L2/3 of the somatosensory cortex led to a significant decrease of the delay time from the start of the Pre-PC AP firing until the first AP in the postsynaptic MC (Fig. 6b, c). In addition, the number of APs fired by the postsynaptic MC following ACh application increased significantly (Fig. 6b, c). However, we observed no change of the AP threshold potential or of the frequency of AP firing. These results show that ACh reduces the delay time for the first AP in MCs, which can explain the advanced disynaptic inhibition upon ACh release, as well as an increase in the number of APs fired, which can account for the increase in time course and amplitude of the lateral inhibition.

### Lateral inhibition in human temporal cortex

**Fig. 7:**
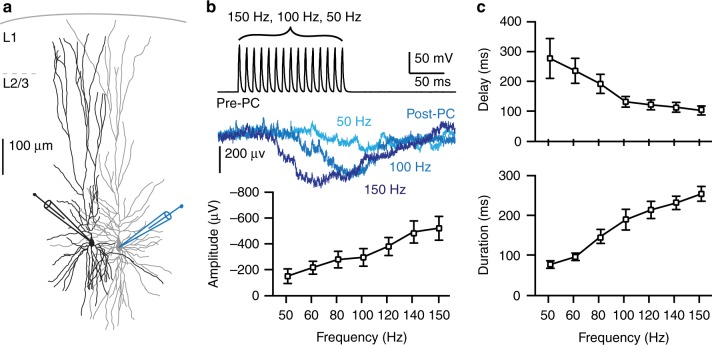
Lateral inhibition between pyramidal neurons in human temporal cortex. **a** Digital reconstruction of two biocytin-filled L2/3 pyramidal neurons in human temporal cortex. **b** Lateral inhibition between two pyramidal neurons in layer 2/3 of the human temporal cortex. Top trace: The Pre-PC fires 15 APs at 100 Hz. Middle traces: Example traces of disynaptic inhibitory responses in the Post-PC neuron following Pre-PC APs at 50, 100, and 150 Hz (50 Hz *n* = 4, 60–100 Hz *n* = 5, 120–150 Hz *n* = 6). (Bottom) Summary plot showing that the amplitude of lateral inhibition increased depending on the Pre-PC AP frequency (50 Hz 0.14 ± 0.05 mV, 100 Hz 0.29 ± 0.06 mV, 150 Hz 0.52 ± 0.09 mV, mean ± s.e.m.). **c** Summary plots showing that lateral inhibition decreased in latency (Top panel, 50 Hz 277.2 ± 66.98 ms, 100 Hz 131.5 ± 17.83 ms, 150 Hz 102.1 ± 15.08 ms) and increased in duration (Bottom panel, 50 Hz 76.04 ± 9.36 ms, 100 Hz 189.2 ± 26.21 ms, 150 Hz 254.1 ± 18.63 ms) depending on the firing frequency of the Pre-PC,(mean ± s.e.m.)

The concept of lateral inhibition between pyramidal neurons is reported in rat and mouse cortices^5,33^. However, it is not known whether this mechanism also exists in the human brain. To test this, we simultaneously recorded from up to four neighboring L2/3 pyramidal neurons in acute human neocortical slices (Fig. 7a) from temporal cortex tissue resected during surgical treatment of epilepsy or tumor patients to gain access to deeper structures^34–37^. Electrical stimulation of pyramidal neurons (Pre-PC) to induce 15 APs at different frequencies (50–150 Hz) induced characteristic lateral inhibition in postsynaptic pyramidal neurons (Post-PC) (Fig. 7b, blue traces). With increasing AP frequency fired by Pre-PCs, the onset delay decreased, the duration of the inhibition increased as well as the amplitude (Fig. 7b, c). Presynaptic electrical stimulation with a frequency below 50 Hz rarely resulted in lateral disynaptic IPSPs in postsynaptic pyramidal neurons. Our results indicate that disynaptic lateral inhibition exists between pyramidal neurons in layer 2/3 of the human neocortex.

**Fig. 8:**
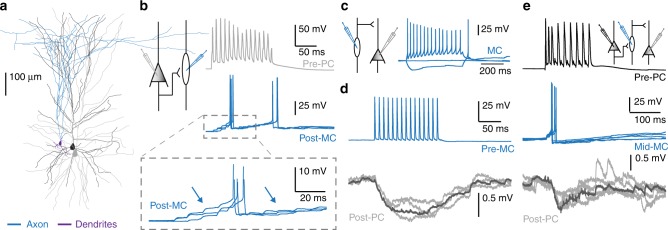
Lateral inhibition in the human cortex is mediated by putative Martinotti interneurons. **a** Digital reconstruction of a complete disynaptic loop in human neocortex between two biocytin-filled pyramidal neurons and a putative Martinotti interneuron (PC (black)-MC (axon (blue), dendrites (purple)-PC (gray)). **b** Left: Schematic representation of the experiment: a presynaptic pyramidal (Pre-PC) and a postsynaptic low-threshold spiking interneuron (Post-MC) recorded in L2/3 of the human temporal cortex. Right: Example trace from a PC-Pre cell firing 15 APs at 100 Hz (Gray trace) and facilitating EPSPs (blue arrows) in the synaptically connected Post-MC cell (blue trace). **c** Left: Schematic representation of the experiment: simultaneous recording from a presynaptic MC and postsynaptic Post-PC. Right: Example trace showing a firing pattern characteristic of MCs, with rebound spiking, the sag in response to hyperpolarization and spike frequency accommodation in a response to depolarizing current injection. **d** Simultaneous recording of a Pre-MC and a Post-PC in L2/3 of the human temporal cortex. The Pre-MC was stimulated to fire 100 Hz AP trains (15 APs) (Blue trace) resulting in the postsynaptic inhibition in the Post-PC cell (Gray trace, average shown in black). **e** A complete disynaptic lateral inhibitory loop in layer 2/3 of the human temporal cortex. Triggering a train of APs (100 Hz, 15 APs) in the presynaptic pyramidal neuron (Pre-PC, black trace) led to the activation of a facilitating connection in the postsynaptic MC (Mid-MC, blue trace). The excitatory inputs evoked an AP in the mid-MC that resulted in IPSPs in the synaptically connected pyramidal neuron (Post-PC, gray trace, average indicated in black)

Since lateral inhibition in the neocortex of rodents is mediated by somatostatin-positive MCs^3,5,16^, we asked whether this specific inhibitory cell type is also mediating disynaptic inhibitory loops in the human neocortex. In rodents, MCs have unique morphological and cellular properties that distinguishes them from other interneuron types, such as axonal projections to L1 and marked rebound APs^5,38–41^. In our recordings, we found interneurons that share similar morphological and cellular characteristics as MCs in rodent neocortex (Fig. 8)^5,38,41,42^. These human interneurons had axons projecting to L1, a bipolar dendritic morphology where the dendritic tree is significantly smaller than the axonal tree and an oval soma (Fig. 8a). Furthermore, these interneurons also had a low spiking threshold with a prominent rebound spike (Fig. 8c), responded with a sag to hyperpolarizing current steps and showed AP accommodation to depolarizing current steps (Fig. 8c). Excitatory EPSPs from Pre-PCs showed facilitation and summated to supra-threshold AP firing (Fig. 8b, blue arrows). These findings indicate that in layer 2/3 of the human cortex, low-threshold spiking cells exist that share numerous morphological and physiological characteristics with MCs in rodents. In addition, when recording from all three components of a disynaptic lateral inhibition loop in human L2/3 we found the same features of disynaptic lateral inhibtion as described in rodent cortex:^5^ as in rodent cortex, high frequency AP firing (100 Hz, 15 APs) by presynaptic pyramidal neurons (Pre-PC), (Fig. 8e black trace) led to facilitating EPSPs in low-threshold spiking interneurons, which in its turn, resulted in AP firing (Fig. 8e blue trace) that caused time-locked IPSPs in the postsynaptic pyramidal neuron (Fig. 8e gray trace). Our findings show that disynaptic lateral inhibition exists in the human neocortex and is mediated by low-threshold spiking interneurons that share several features with rodent MCs.

### Acetylcholine enhances lateral inhibition by activating nAChRs in human temporal cortex

**Fig. 9:**
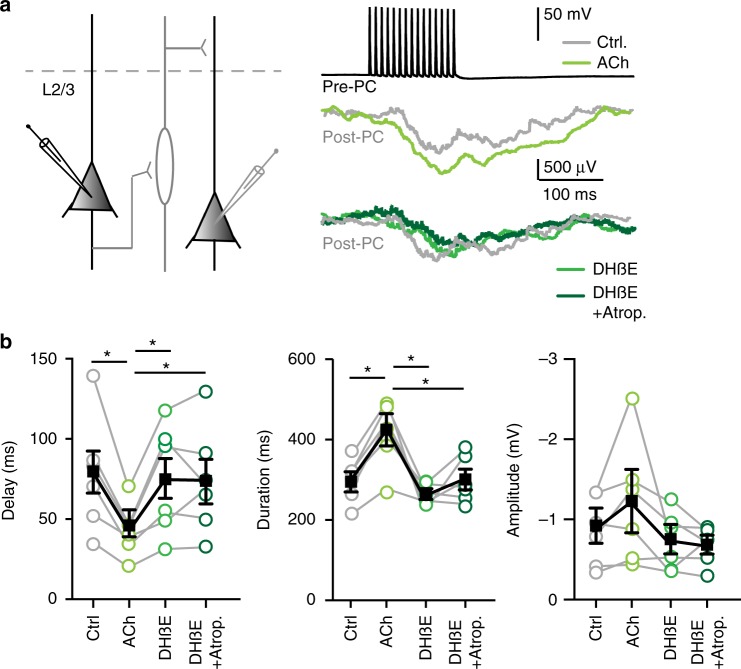
ACh facilitates lateral inhibition in human neocortex. **a** Left: schematic illustration of the experiment: simultaneous recording of pyramidal neurons in L2/3 of the human temporal cortex showing lateral inhibition. Right: top trace: 15 APs at 100 Hz (black trace) in the Pre-PC. Middle trace: Example trace of lateral inhibitory response in the Post-PC neuron in ACSF (gray trace) or in presence of ACh (1 mM) (green trace). Bottom trace: as middle traces, ACh (1 mM) was bath applied in presence of DHßE (10 µM) (light green trace) or DHßE (10 µM) and Atropine (400 nM) (dark green trace). **b** Summary plots showing that the onset delay time of lateral inhibition in the Post-PC is decreased (F_(5, 15)_ = 24,37, *p* = 0.001; Two-way ANOVA, *n* = 6, mean ± s.e.m.) and the duration increased following ACh application F_(5, 15)_ = 4.669, *p* = 0.009; Two-way ANOVA, *n* = 6). The heteromeric nAChR blocker DHßE blocks these effects. The combined bath application of DHßE and atropine had no additional effect on lateral inhibition. The amplitude was not affected by ACh (F_(5, 15)_ = 7.153, *p* = 0.09; Two-way ANOVA, *n* = 6, mean ± s.e.m.)

Since ACh is facilitating disynapic lateral inhibition in rodent neocortex, we asked whether cholinergic modulation of lateral inhibition is conserved in human cortex. To test this, we induced lateral inhibition by electrically stimulating presynaptic pyramidal neurons (Pre-PC) to fire 15 APs with 100 Hz while recording IPSPs in the postsynaptic pyramidal cell (Post-PC, Fig. 9a). Following wash-in of ACh (1 mM), the onset delay time of disynaptic IPSPs in Post-PCs was reduced and the duration of inhibition increased, while the amplitude was not affected (Fig. 9a, b). Bath application of DHßE (10 µM) blocked these effects by ACh (Fig. 9a, b, light green trace). Blocking muscarinic acetylcholine receptors by bath application of atropine (400 nM) did not affect the onset, duration or amplitude of lateral IPSPs (Fig. 9a, b, dark green trace). We observed no additional effects on lateral inhibition between pyramidal neurons following combined bath application of DHßE and atropine. These findings suggest that lateral inhibition in L2/3 of the human temporal neocortex is facilitated by activation of heteromeric nAChRs and not muscarinic acetylcholine receptors.

### ACh depolarizes human putative Martinotti cells and alters AP firing properties

**Fig. 10:**
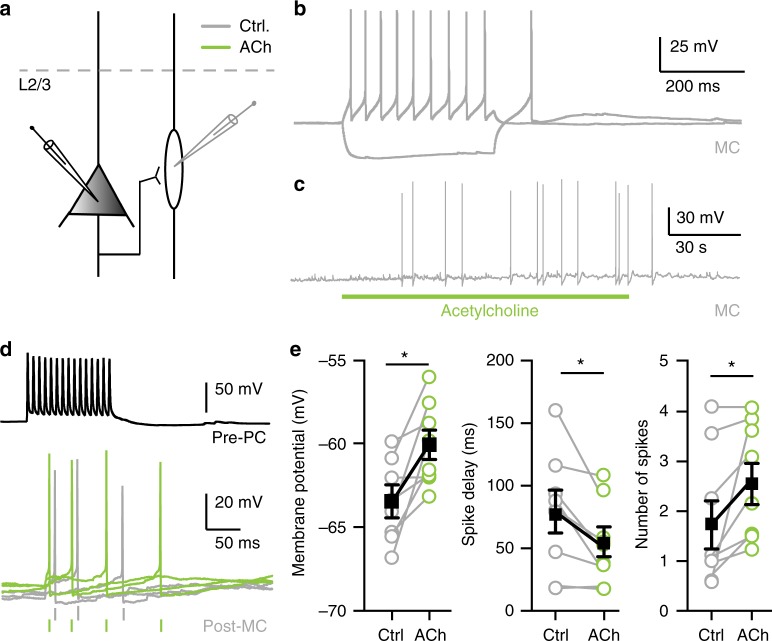
ACh facilitates AP firing by human putative Martinotti cells. **a** Schematic illustration of the experiment: recording of synaptically connected human pyramidal (Pre-PC) and putative Martinotti cell (Post-MC). **b** Spiking profile of the Post-MC. **c** Example trace showing that Post-MCs can start to firing APs upon ACh (1 mM) application (*n* = 2 of 8). **d** Recording of synaptically connected human pyramidal cell (Pre-PC) and putative MC (Post-MC). Top-trace: The pyramidal neuron is electrically stimulated to fire a train of 15 APs at 100 Hz. Bottom trace: Typical traces showing the excitatory input in the Post-MC without (gray trace) and with ACh (green trace) application. **e** Summary plots showing that ACh depolarized putative MCs (from −63.54 ± 0.85 mV to −60.15. ± 0.88 mV, *p* = 0.03; paired *t*-test; two-tailed; *t* = 3.699, df = 7; *n* = 8, mean ± s.e.m.), decreased the spike delay (from 76.12 ± 17.51 ms to 51.43 ± 12.21, *p* = 0.023; paired *t*-test; two-tailed; *t* = 2.89, df = 7; *n* = 8, mean ± s.e.m.) to the first spike and increased the number of APs (from 1.713 ± 0.48 APs to 2.531 ± 0.41 APs, *p* = 0.045; paired *t*-test; two-tailed, *t* = 2.427, df = 7; *n* = 8, mean ± s.e.m.). The spiking threshold and firing frequency was not affected by ACh (*p* = 0.439, paired *t*-test; two-tailed, *t* = 0.8199, df = 7, *n* = 8; *p* = 0.094, paired *t*-test; two-tailed, *t* = 2.185, df = 4, *n* = 5, mean ± s.e.m.)

As we showed above, cholinergic facilitation of lateral inhibition in rodent neocortex is mediated by heteromeric nAChRs that depolarize MCs. Since interneurons in the human neocortex express also various types of nAChRs^43,44^, we asked whether depolarization of putative MCs by ACh mediates cholinergic facilitation of lateral inhibition in human neocortex. To test this, we recorded from human putative MCs in L2/3 (Fig. 10a), identified by the morphological and electrophysiological criteria described above (Fig. 10b). Bath application of ACh (1 mM) for 15 min depolarized these neurons (Fig. 10c, e). In 2 out of 8 recordings, ACh application induced spontaneous AP firing in putative MC (Fig. 10c). This suggests that somatic depolarization of putative MC interneurons may mediate facilitation of lateral inhibition in the human cortex.

To test whether ACh modulates AP firing in putative MCs during lateral inhibition, we simultaneously recorded from synaptically connected pyramidal neurons and putative MCs in L2/3 of the human temporal cortex. We electrically stimulated the presynaptic pyramidal cell (Pre-PC) to fire 15 APs at a frequency of 100 Hz and recorded AP firing by the postsynaptic putative MC. Recordings were only included for analyses in which the postsynaptic interneuron reliably fired APs following presynaptic stimulation in all experimental conditions. Presynaptic stimulation combined with ACh application (1 mM) led to a shorter latency of the first AP (Fig. 10d, e) and more APs in the postsynaptic interneuron compared to presynaptic stimulation alone (Fig. 10d, e). The AP threshold potential and frequency of APs in the postsynaptic interneuron was not changed. Our findings show that ACh depolarizes human putative MCs mediated by heteromeric nAChRs, advancing both AP firing in MCs and lateral inhibition between human pyramidal neurons.

## Discussion

In this study, we addressed the question whether cholinergic projections from the basal forebrain modulate cortical lateral inhibition between pyramidal neurons. We find that (1) in L2/3 and L5, optogenetic activation of mainly BF projections shortens the delay time and increases the duration of delayed lateral inhibition via Martinotti cells of neighboring pyramidal neurons, while fast lateral inhibition is not affected by cholinergic inputs. (2) Cholinergic facilitation of lateral inhibition is independent of firing frequencies of presynaptic pyramidal neurons. (3) We show that heteromeric nAChRs containing ß2 subunits, rather than muscarinic AChRs, mediate this cholinergic modulation. (4) The mechanism of cholinergic facilitation of lateral inhibition between pyramidal neurons relies on direct depolarization of MCs mediated by postsynaptic heteromeric nAChRs. Co-occurrence of glutamatergic and cholinergic excitatory inputs summate linearly. Strength of synapses between pyramidal cells and MCs is not affected by ACh. (5) ACh leads to a significant decrease of the onset delay of AP firing and increases the number of AP fired in MCs, which can account for the earlier onset and prolonged duration of disynaptic inhibition. (6) In addition, we show that delayed disynaptic lateral inhibition between pyramidal neurons is conserved in the human cortex and is modulated by putative MCs. (7) In the human cortex, mechanisms of cholinergic modulation of lateral inhibition are similar to rodent cortex. In short, cholinergic inputs selectively augment disynaptic lateral inhibition via MCs in rodent and human cortex by increasing excitability of MCs.

Pyramidal neurons in the cortex can suppress activity of surrounding pyramidal neurons through lateral inhibition mediated by MCs and PV-positive interneurons^3–5^. Excitatory synapses between pyramidal neurons and most types of interneurons are depressing, but excitatory synapses between pyramidal neurons and SOM-positive MCs are facilitating^3–5,8,12,45^. With increasing firing frequencies of pyramidal neurons, stronger synaptic facilitation occurs in MCs and the probability of generating action potentials increases. As a consequence, higher firing frequencies of presynaptic pyramidal neurons speed up the discharge of MCs, which leads to earlier onset of AP firing and more APs^5,46^. Glutamatergic synapses can be facilitated by nAChRs located on presynaptic glutamatergic terminals^30–32^, and BF cholinergic inputs can alter the strength of glutamatergic synapses in a layer-dependent fashion^37^. Furthermore, depolarization of presynaptic pyramidal cells can increase glutamatergic facilitation in the pyramidal cell to MC synaptic pathway, which augments lateral inhibition between pyramidal neurons^47^. In our experiments, ACh did not affect the membrane potential of presynaptic pyramidal neurons or alter presynaptic facilitation of EPSPs between pyramidal and MCs. We did find that cholinergic inputs depolarize MCs, giving rise to an earlier onset and a higher number of APs in MCs. Furthermore, we showed that cholinergic inputs speed up the onset and increase the duration of disynaptic inhibition in neighboring pyramidal neurons. The increased amplitude of disynaptic IPSPs following optogentically triggered release from cholinergic inputs but not bath applied ACh in the mPFC might therefore be the result of a larger number of MCs that reach firing threshold and take part in lateral inhibition. Since bath application of ACh can lead to desensitization of nicotinic receptors it is most likely that the observed discrepancy in the modulation of the amplitude is caused by the difference between optogenetically triggered and ACh bath application. Disynaptic inhibition produced by MCs can affect a substantial fraction of neighboring pyramidal neurons as a result of high connection probability between MCs and pyramidal neurons, reported in both juvenile and adult rodent neocortex^2,16,48^. By depolarizing MCs, cholinergic inputs can dynamically facilitate recruitment of MCs by pyramidal neurons to take part in lateral inhibition.

Synchronized firing by pyramidal neurons is controlled by interneuron activity^1,28^. Lateral inhibition mediated by Martinotti cells can synchronize and maintain firing activity of pyramidal neurons in the cortex^28^. Since ACh facilitates delayed lateral inhibition mediated by MC, cholinergic signaling may amplify the synchronization of the firing behavior of pyramidal neurons.

Lateral inhibition between pyramidal neurons is specifically mediated by MCs, a specific subtype of SOM expressing interneurons^1,5^. Recently it was reported that in vivo, synapses between pyramidal neurons activated at low frequencies and SOM neurons have a high failure rate that is reduced by ACh acting via a PKA signaling pathway^49^. In contrast, we found that synapses from pyramidal neurons to MCs mediating disynaptic lateral inhibition, are not affected by ACh application or optogenetically induced ACh release. Since lateral inhibition is specifically mediated by MCs^5^ and Urbano-Ciecko et al.^49^ did not focus on a specific SOM interneuron subtype, it is possible that ACh differentially modulates synapses from pyramidal neurons to subtypes of SOM interneurons.

Fast lateral inhibition is mediated by PV-positive interneurons^5^. A subgroup of these interneurons express fast nAChR currents mediated by α7-containing nAChRs in mPFC L5^11^. These nicotinic currents act on PV interneurons on a time scale similar to currents acting at glutamatergic synapses, and they can be activated by optogenetic activation of basal forebrain ACh inputs^50^. We showed that fast lateral inhibition is not affected by cholinergic projections. Possibly in lateral inhibition, PV-positive interneurons are recruited that do not express nAChRs^11^. PV-positive interneurons target perisomatic regions of pyramidal neurons, and are well-suited to control timing of action potentials, whereas SOM-positive MCs target distal areas of pyramidal neuron dendrites, affecting dendritic integration^4,6,51,52^. Since lateral inhibition via MCs is ACh sensitive, cholinergic inputs can selectively alter inhibitory pathways between pyramidal neurons, shifting the balance between somatic and dendritic processing.

Cholinergic receptors are widely distributed among different cell types in the cortex^11,22,44,53^. In the neocortex, optogenetic ACh release from projections predominantly activate nicotinic AChR currents^24,26,37,54,55^. A prominent feature of SOM-positive interneurons is the strong membrane depolarization caused by agonists of both muscarinic and nicotinic AChRs^8–13^. ACh from the basal forebrain could in principle activate both types of receptors expressed by MCs. In the thalamus, endogenous release of ACh by optogenetic stimulation results in a biphasic response caused by activation of both nicotinic and muscarinic ACh receptors^56^. We did not observe this in MCs. Furthermore, cholinergic projections induced a prominent depolarization in MCs, which was completely abolished by nicotinic AChR blockers. This suggests that although MCs express muscarinic AChRs, cholinergic inputs preferably activate nicotinic AChRs and not muscarinic AChRs.

The activation of nAChRs by cholinergic inputs does not have to reach firing threshold by itself, unlike in the visual cortex, where supra-threshold cholinergic recruitment of SOM-positive interneurons alters local network activity to a more desynchronized state^13^. Cholinergic modulation of lateral inhibition by cholinergic inputs can occur without supra-threshold cholinergic activation of the SOM-positive interneurons. Sub-threshold depolarization by cholinergic inputs is sufficient to facilitate lateral inhibition between pyramidal neurons, and advance action potential firing of SOM-positive interneurons induced by pyramidal neuron inputs.

Recently, several studies highlighted similarities and differences in cellular and synaptic function between rodent and human neocortical circuitry^34,35,37,57–61^. Although inhibition mediated by fast-spiking interneurons is described^57^, we found here that layer 2/3 pyramidal neurons in the human cortex modulate activity of surrounding pyramidal neurons through delayed lateral inhibition mediated by putative MCs. Although it was reported that single AP firing in the presynaptic pyramidal neuron can trigger complex events in the human cortex^57,61^, we did not observe complex events or fast lateral inhibition between pyramidal neurons in our recordings. We did find some variation in amplitudes of disynaptic IPSPs in human pyramidal neurons. Possibly, this results either from variation in the number of MCs that are recruited by presynaptic pyramidal neurons, or variation in the strength of synapses involved. Nevertheless, we found consistently that in the presence of ACh, the amplitude of IPSP amplitudes was increased. Given that MCs are depolarized by ACh while synaptic strength is unchanged, this suggests that the likelihood of MCs being recruited was increased.

Various reports show that fast cholinergic signaling plays an important role in modulating cellular activity and microcircuits in the rodent brain^11,62^. However, little is known about whether cholinergic modulation of information processing in the human neocortex follows similar mechanisms. EM studies show that 67% of all varicosities on cholinergic axons in the human temporal cortex can be identified as point-to-point synapses, in contrast to only 15% in rodent cortex, which suggests that in human neocortex fast cholinergic signaling may be more abundant^63^. Pyramidal neurons and interneurons in the human cortex express α7-containing and β2-containing nAChRs acting on a fast time scale^37,43,44^. Our results show that ACh facilitates the onset and duration of delayed disynaptic lateral inhibition by activating heteromeric nAChRs. Cholinergic depolarization, advancement of spiking onset, and higher spiking rate of putative MCs appear to be conserved in human neocortex.

Cholinergic modulation of interneurons is important for cortical processing^10,13,17–19,64^. For example, disinhibitory pathways activated by cholinergic inputs to interneurons in superficial layers of the auditory cortex control auditory fear conditioning^65^. Whether similar mechanisms are in place in the mPFC is not known. However, cholinergic control of mPFC circuits is behaviorally relevant. In mice lacking nAChR ß2 subunit attentional performance is reduced^66^, and during attention behavior the amount of ACh in the mPFC rapidly increases to make a shift from monitoring cues towards a cue evoked goal directed response^67,68^. Lateral inhibition and its modulation may also be important during sensory processing since MCs in somatosensory cortex are activated during whisking in a layer-specific manner^69^. Thereby, modulation of lateral inhibition may serve sensory and cognitive processes to enhance, on demand, signal-to-noise ratio in pyramidal neuron activity.

## Methods

### Mouse brain slice preparation

Coronal medial prefrontal cortex (mPFC) or parasaggital somatosensory (S1) slices were prepared from P14–25 male or female C57Bl/6 mice, Gin mice [FVBTg(GadGFP)45704Swn/J from the Jackson Laboratory]^29^ or the F1 of matings between Gin mice with Chat-Chr2-EYFP mice [B6.Cg-Tg(Chat-COP4*H134R/EYFP)6Gfng/J from the Jackson Laboratory (Jax, USA). Following decapitation, the brain was carefully removed from the skull and maintained and sliced in carbogen buffered (95 % O2, 5 % CO2 at pH 7.4) ice-cold slicing solution containing (in mM): 125 NaCl, 3 KCl, 1.25 NaH_2_PO_4_, 7 MgSO_4_, 0.5 CaCl_2_, 26 NaHCO_3_, and 10 glucose. Acute brain slices (350 µm) were incubated for 1 min at 34 °C in N-Methyl-d-glucamin solution (NMDG solution; in mM: NMDG 93, KCl 2.5, NaH_2_PO_4_ 1.2, NaHCO_3_ 30, HEPES 20, Glucose 25, NAC 12, Sodium ascorbate 5, Sodium pyruvate 3, MgSO_4_ 10, CaCl_2_ 0.5, at pH 7.4 adjusted with 10 M HCl). For recovery, slices were maintained at room temperature in artificial cerebrospinal fluid (aCSF) containing (in mM) 125 NaCl, 3 KCl, 1.25 NaH_2_PO_4_, 1 MgSO_4_, 2 CaCl_2_, 26 NaHCO_3_, and 10 glucose in a holding chamber for at least 1 h prior to recordings.

### Human brain slice preparation

All performed procedures on human tissue were in line with the Dutch license procedures and the declaration of Helsinki and approved by the Medical Ethical Committee of the VU University Medical Centre. To reach deeper brain regions for surgical treatment, human anterior and medial temporal cortex had to be removed. For this study, we obtained tissue from the temporal lobe from five patients (three females, two males, aged 32–52 years) with written informed consent. The dissected temporal tissue showed no abnormalities on preoperative MRI and was classified by neuropathologists as non-pathological. All patients were diagnosed with meso-temporal epilepsia and had mild to severe forms of epilepsy.

Slice preparation from human brain tissue followed a procedure that was developed in our lab^34–37,58,59^. Resected cortical tissue blocks from the temporal cortex were transported and sliced in ice-cold choline slicing solution containing (in mM): 110 choline chloride, 26 NaHCO_3_, 10 d-glucose, 11.6 sodium ascorbate, 7 MgCl_2_, 3.1 sodium pyruvate, 2.5 KCl, 1.25 NaH_2_PO_4_ and 0.5 CaCl_2_. The slice preparation started maximal 10 min after the tissue resection. In a first step the pia was removed from the tissue block using fine forcipes and the pia-white matter (WM) axis were identified. Then the tissue block was fixated on a platform and 350-µm-thick brain slices were prepared using a Thermo scientific slicer (Microm HM 650 V) in ice-cold choline slicing solution. Human cortical slices were transferred to a holding chamber with aCSF for 30 min at 34 °C. Subsequently the slices were incubated for recovery at room temperature for at least one hour before starting recordings. The recordings were performed in aCSF at 32 °C and a flowrate of 2–3 mL/min.

### Electrophysiology

Simultaneous whole-cell recordings from up to four connected pyramidal (PC) and Martinotti cells (MCs) in L5 of the mPFC or L2/3 of the somatosensory cortex (S1) or slices from the human temporal cortex were made in oxygenated aCSF (flow rate of 3–4 mL/min, 32 °C). For recordings, boroscilicate glass pipettes (3–6 MΩ) filled with a potassium based internal solution (in mM): K-gluconate 135, NaCl 4, HEPES 10, Mg-ATP 2,K2Phos 10, GTP 0.3, EGTA 0.2 were used. The recorded values were not corrected for junction potential. The estimated junction potential is 16.3 mV. Pyramidal neurons were identified under the DIC by the typical triangle shape and following untargeted patch by their spiking profile. MCs were identified in the GIN mice by expression of GFP^29^, spike profile, and bipolar morphology. We minimized the exposure to blue light to avoid long lasting activation of ChR2 and let the tissue recover for at least 5 min before recording. In recordings without MCs, there was no exposure to blue light preceding the recordings. During recordings, PC and MCs were kept at holding membrane potentials close to −60 mV. To quantify disynaptic or monosynaptic connections, presynaptic neurons were injected with 2 nA pulses of 2 ms to evoke a train of 15 APs at a frequency of 100 Hz with an inter-train interval of 7 s. Following electrical stimulation in 23.36% and in 0.4% of the recordings were respectively delayed disynaptic inhibitory loops or fast disynaptic inhibitory loops observed between layer 5 pyramidal neurons. Fifteen APs per train were also used for experiments where presynaptic cells were stimulated to fire AP trains at different frequencies. In multiple cell recordings, each cell was stimulated with an interval of 60 s in an alternating manner. The postsynaptic excitatory or inhibitory response were analyzed and quantified by averaging 5–20 traces. Amplitudes were calculated as the difference between the peak value and the average baseline of 100 ms before the stimulation onset. The onset latency was calculated from the start of the stimulation in the presynaptic cell to the threshold of the response at 20% of the maximum amplitude, response duration was calculated as the time difference between the onset threshold and the offset threshold at 20% of the maximal amplitude.

### Optogenetically evoked endogenous acetylcholine release

In Gin/Chat-ChR2-EYFP-crossed mice, cholinergic fibers were stimulated by blue light activation of channelrhodopsin (ChR2) (five light pulses, 470 nm, @ 25 Hz) using a DC4100 4- channel LED-driver (Thorlabs, Newton, NJ) or a Fluorescence lamp (X-Cite Series 120q, Lumen Dynamics). In experiments where light stimulation was combined with presynaptic electrical stimulation the first light pulse started 100 ms before the first AP in the presynaptic neuron. The presynaptic stimulation was either with light off or with light on, alternating with 60 s interval. In some experiments we observed feedforward inhibitory responses by blue light, as was reported previously^12^. These recordings were excluded from analysis. In layer 5, we sometimes observed feedforward excitatory responses by blue light, which was prevented by reducing the field of illumination.

### Pharmacology

All drugs used were dissolved in aCSF at the final concentration and bath applied during the experiments. Drug concentrations used were: acetylcholine (1 mM, Sigma-Aldrich), atropine (400 nM; Sigma-Aldrich), DHßE (10 µM; Tocris Bioscience). All experiments were performed without application of synaptic blockers.

### Biocytin staining and example images of neurons

Recorded cells were filled with biocytin that was added to the intracellular solution (5 mg/mL). Following recordings, slices were fixated in 4% paraformaldehyde for 24 h and then transferred to 1× PBS. Recorded cells were made visible by using chromogen 3,3-diaminobenzidine tetrahydrochloride with the avidin-biotin-peroxidase method^70^. After mounting in mowiol (Clariant GmbH, Frankfurt am Main, Germany) images of visible neurons were made using a ×40 oil objective. Example images of neurons in Figs. 1, 5–10 were made by reconstruct the neurons using adobe illustrator.

### Cluster analysis

To distinguish between fast and delayed lateral inhibition a K-means cluster analyses were performed using MATLAB (R2017b). The clusters were formed based on the variables, Delay and Time to peak(Supplementary Figure 1).

### Analysis and statistics

Raw data was analyzed using Clampfit 10.4. or custom-written Igor Pro scripts (Igor Pro 7 waveMetrics). The junction potential was calculated using pCLAMP (version 10.7.0.). Statistical analysis was performed using Prism 6 (GraphPad software). The D’Agostino–Pearson omnibus normality test was used to investigate whether the data was normally distributed. Statistical tests used to evaluate the data are mentioned in the figure legends, data shown are mean ± s.e.m., and *p* < 0.05 was taken as level of significance.

## Electronic supplementary material

Supplementary Information

## Data Availability

The data that support the findings of this study are available from the corresponding author on request.
