# Supplementary material for: Lateral inhibition by Martinotti interneurons is facilitated by cholinergic inputs in human and mouse neocortex
Source: Nat Commun. 2018 Oct 5;9:4101. doi: 10.1038/s41467-018-06628-w (PMC6173769; doi:10.1038/s41467-018-06628-w)
Supplement: Supplementary file 1 — Supplementary Information [file 41467_2018_6628_MOESM1_ESM.pdf]

Lateral Inhibition by Martinotti Interneurons is Facilitated by Cholinergic Inputs in Human and Mouse Neocortex

Obermayer et al.

Supplementary Figure 1. K-means cluster analysis

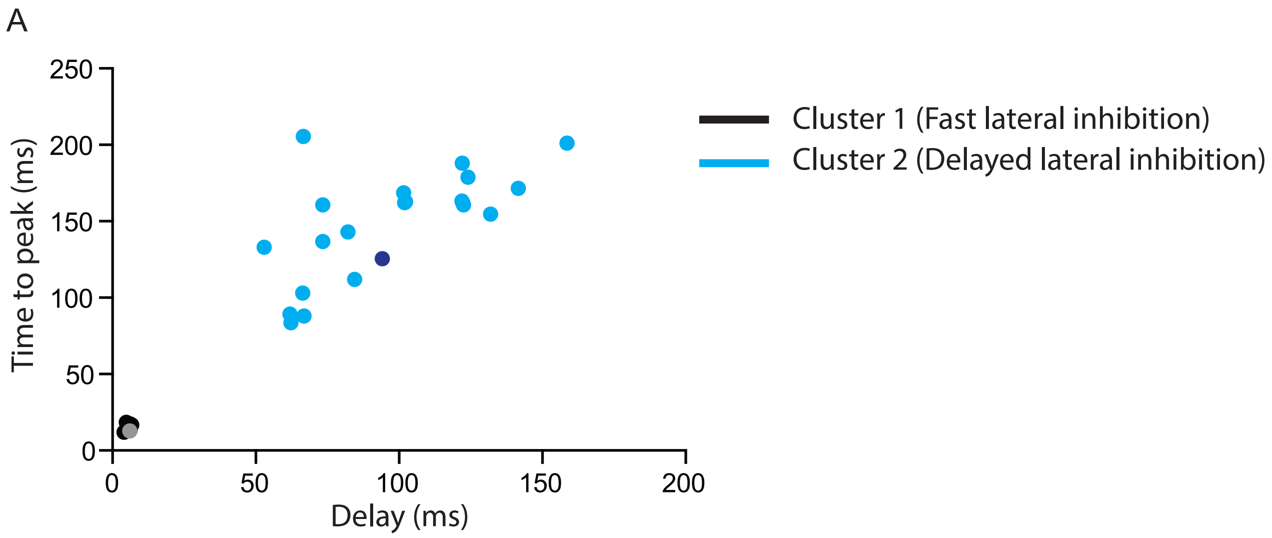

A) Two clusters of disynaptic IPSPs identified by K-means cluster analyses (C= 5.582, 15,01; sumd=43.3525). The two clusters are clearly distinguishable and represent fast (cluster 1, the grey dot indicates the data from Fig. 1D) and delayed lateral inhibition (cluster 2, the purple dot indicates the data from Fig. 1C).

Supplementary Figure 2. Bath application of ACh facilitates lateral inhibition.

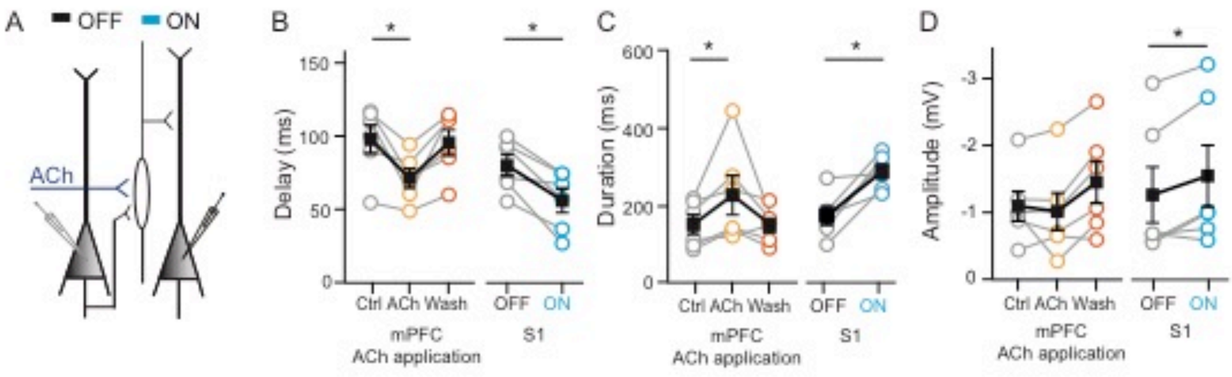

A) Schematic illustration of the experiment showing a recording from two pyramidal neurons in S1.

B) Summary chart showing that ACh shortens the onset delay of lateral inhibition. Bath application of ACh leads to decreased onset latency (Ctrl. 96±11 ms, ACh 77±6 ms, wash 90±8 ms, paired *t*-test, two-tailed, $p<0.05$, $t=2.895$, $df=5$, $n=6$, mean±s.e.m.) similar to BF cholinergic projection activation. In somatosensory cortex(S1) cholinergic inputs decrease the onset delay of lateral inhibition (light OFF 81±7 ms, light ON 56±8 ms ; paired *t*-test, two-tailed, $p<0.05$, $t=4.5$, $df=5$, $n=6$, mean±s.e.m.).

C) As in (**B)** showing that ACh increases the duration of lateral inhibition. Bath application of ACh leads to an increase in the duration (Ctrl. 148±24 ms, ACh 223±50 ms, wash 145±18 ms, Wilcoxon signed-rank test, $p<0.05$, $n=6$, mean±s.e.m). In S1 we observed also a modulation of duration of the lateral inhibition (light OFF 171±23 ms, light ON 284±18 ms, Wilcoxon signed-rank test, $p<0.01$, $n=6$, mean±s.e.m)

D) As in (**B)** and (**C)** showing that ACh increases the amplitude of lateral inhibition in S1. Bath application: paired *t*-test, two tailed, $p=0.3648$, $t=0.9963$, $df=5$, $n=6$; S1: light OFF 1.27±0.42 mV, light ON 1.56±0.46 mV; paired *t*-test, two-tailed, $p<0.05$, $t=2.8$, $df=5$, $n=6$, mean±s.e.m.).

Supplementary Figure 3. Endogenous ACh does not affect the synaptic strength PCs and MCs.

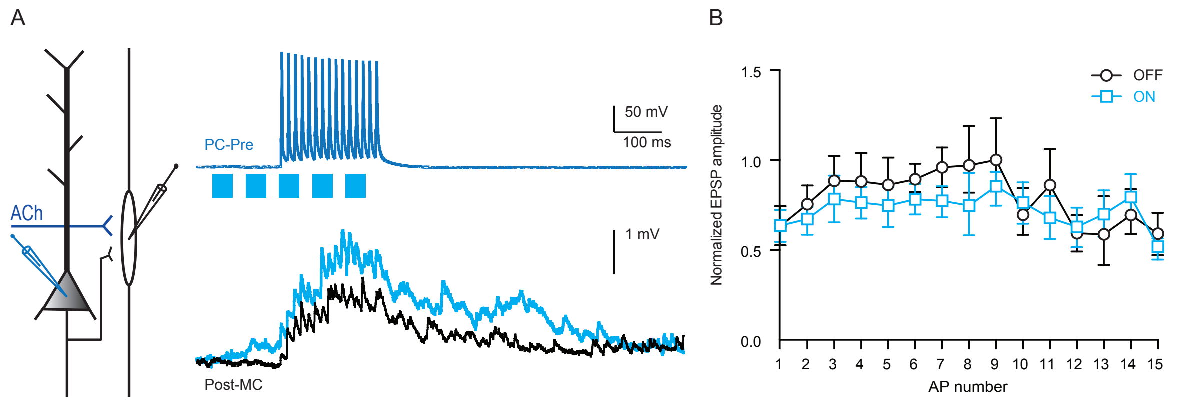

A) Left: schematic representation of the simultaneous recording of a presynaptic pyramidal cell (Pre-PC) and a postsynaptic Martinotti cell (Post-MC). Example trace recorded from a PC-Pre cell injected with current to evoke 15 APs at a frequency of 100 Hz (Blue trace) and the EPSPs in the Post-MC cell (Black trace).

B) Summary plot of the normalized amplitude of EPSPs recorded in post-MCs. The amplitude was normalized to the EPSP with the in average largest amplitude. Endogenous ACh released from cholinergic fibers by applying blue light pulses did not alter synaptic strength between the Pre-PC and Post-MC ($F_{(14, 180)}$=1.627, p=0.0756 Two-Way ANOVA; n=7 mean±s.e.m.).
